# Planetary Stewardship in an Urbanizing World: Beyond City Limits

**DOI:** 10.1007/s13280-012-0353-7

**Published:** 2012-10-18

**Authors:** Sybil P. Seitzinger, Uno Svedin, Carole L. Crumley, Will Steffen, Saiful Arif Abdullah, Christine Alfsen, Wendy J. Broadgate, Frank Biermann, Ninad R. Bondre, John A. Dearing, Lisa Deutsch, Shobhakar Dhakal, Thomas Elmqvist, Neda Farahbakhshazad, Owen Gaffney, Helmut Haberl, Sandra Lavorel, Cheikh Mbow, Anthony J. McMichael, Joao M. F. deMorais, Per Olsson, Patricia Fernanda Pinho, Karen C. Seto, Paul Sinclair, Mark Stafford Smith, Lorraine Sugar

**Affiliations:** 1 International Geosphere Biosphere Programme, Royal Swedish Academy of Sciences, Box 50005, 104 05 Stockholm, Sweden; 2 Stockholm Resilience Centre, 106 91 Stockholm, Sweden; 3 Department of Archaeology and Ancient History, Uppsala University, 75126 Uppsala, Sweden; 4 Center for Biodiversity, Swedish Agricultural University, Box 7007, 750 07 Uppsala, Sweden; 5 The ANU Climate Change Institute, The College of Asia and the Pacific, Australian National University, Coombs Building, Canberra, ACT 0200 Australia; 6 Institute for Environment and Development (LESTARI), Universiti Kebangsaan Malaysia, 43600 Bangi, Selangor Darul Ehsan Malaysia; 7 Chemin du Vier, 06380 Sospel, France; 8 Institute for Environmental Studies (IVM), VU University Amsterdam, De Boelelaan 1087, 1081 HV Amsterdam, The Netherlands; 9 Geography and Environment, University of Southampton, Southampton, SO17 1BJ UK; 10 Energy Field of Study, Asian Institute of Technology, PO Box 4, Klong Luang, Pathumthani, 12120 Thailand; 11 Swedish Secretariat for Environmental Earth System Sciences, The Royal Swedish Academy of Sciences, Box 50005, 104 05 Stockholm, Sweden; 12 Institute of Social Ecology Vienna, Alpen-Adria Universität Klagenfurt, Schottenfeldgasse 29, 1070 Vienna, Austria; 13 Laboratoire d’Ecologie Alpine, CNRS UMR 5553, BP 53, 2233 Rue de la Piscine, 38041 Grenoble Cedex 9, France; 14 Institut des Sciences de l’Environment, Laboratoired’Enseignementet de Recherche en Géomatique (LERG), Ecole Supérieure Polytechnique (ESP)/FST, Université Cheikh Anta Diop, Dakar, Senegal; 15 National Centre for Epidemiology and Population Health, Australian National University, Canberra, ACT 0200 Australia; 16 IGBP Regional Office Brazil, Brazil Instituto Nacional de Pesquisas Espaciais, São José dos Campos, Brazil; 17 Yale School of Forestry and Environmental Studies, Yale University, 195 Prospect Street, New Haven, CT 06511 USA; 18 CSIRO Climate Adaptation Flagship, Canberra, ACT 2601 Australia; 19 The World Bank Group, 1818 H Street NW, Washington, DC 20433 USA; 20 Present Address: FORSK, Swedish International Development Cooperation Agency, Valhallavägen 199, 105 25 Stockholm, Sweden

**Keywords:** Urban, Rural, Resources, Sustainability, Planetary stewardship, Global, Governance

## Abstract

Cities are rapidly increasing in importance as a major factor shaping the Earth system, and therefore, must take corresponding responsibility. With currently over half the world’s population, cities are supported by resources originating from primarily rural regions often located around the world far distant from the urban loci of use. The sustainability of a city can no longer be considered in isolation from the sustainability of human and natural resources it uses from proximal or distant regions, or the combined resource use and impacts of cities globally. The world’s multiple and complex environmental and social challenges require interconnected solutions and coordinated governance approaches to planetary stewardship. We suggest that a key component of planetary stewardship is a global system of cities that develop sustainable processes and policies in concert with its non-urban areas. The potential for cities to cooperate as a system and with rural connectivity could increase their capacity to effect change and foster stewardship at the planetary scale and also increase their resource security.

## Introduction

Human activities now rival or exceed biogeophysical drivers in transforming the planet to the extent that this time in history warrants an epoch of its own, increasingly referred to as “the Anthropocene” (Crutzen and Stoermer 2000; Crutzen 2002; Steffen et al. 2011). Increasing size and urban concentration of world population, coupled with changing lifestyles and associated consumption patterns, have led to unprecedented resource use and waste generation during the twentieth century. This expanding level of demand requires a portfolio of responses that address environmental, social, and economic issues at the planetary scale. The interconnected nature of problems, the multiple scales and rates involved, and the geopolitical constellations make this a formidable yet urgent challenge.

Research approaches as well as governance responses to date have focused largely on single issues (e.g., air pollution, population, climate, water, etc.) and on the search for solutions and treaties that often do not match the magnitude of the problems. In contrast, many issues are interconnected, the drivers and effects cross many space and time scales, and encompass environmental and socio-economic dimensions. In addition, political imperatives and difficulties in assigning and quantifying responsibilities have contributed to lack of action and slow progress.

Here, we build on and extend previous thinking on earth and planetary stewardship (e.g., Steffen et al. 2004, 2011; Chapin et al. 2011). We define planetary stewardship as the active shaping of trajectories of change on the planet, that integrates across scales from local to global, to enhance the combined sustainability of human well-being and the planet’s ecosystems and non-living resources. To support planetary stewardship a coordinated polycentric governance approach is required that is informed by a deeper understanding of the complex, multi-scalar, and interconnected nature of today’s global environmental challenges. Given the increasing importance of urbanization and concomitant pressure on resources, we contend that one of the necessary elements for achieving stewardship is the sustainability of the emerging global system of cities, including their hinterlands.

## The Urban Dimension

Contemporary urbanization differs from the past in its rate, scale, location, and form (Seto et al. 2010). In 1800, when the world population hovered around 1000 million people, the only city with more than a million inhabitants was Beijing (Chandler 1987). By 1900, about 16 cities had crossed this threshold, a number that swelled to 200 at the beginning of this millennium. If the trend continues, by 2025 there will be around 600 cities worldwide with populations of a million or more. By 2100, the global population is projected to be 3000 million more than today, with 70–90 % of people living in urban regions (UN 2011). This increase in urban population is projected to be not only from global population increase but also from immigration from rural areas.

Currently, more than half of the global population lives in urban areas (UN 2011), although urban areas account for only about 2 % of global land surface (Akbari et al. 2009). These are global centers of production and consumption (Seto et al. 2010). By some accounts, more than 90 % of the world’s gross domestic product (GDP) is produced in urban regions (Gutman 2007). Consequently, urban regions, in both developed and developing countries, use a large amount of energy and other resources (Dhakal 2009). Approximately, 70 % of energy-related carbon emissions, 60 % of residential water use, and 76 % of wood used for industrial purposes is attributed to cities globally (Brown 2001; World Energy Outlook 2008).

## Global Flows and Interconnected Issues

With increasing globalization, materials and energy are drawn in great quantities from all over the world—often from large distances to the primarily urban locus of consumption and waste generation. Such distal flows and dependencies provide a global perspective of the more traditional view of the urban–rural nexus. For example, fish meal is imported from marine ecosystems worldwide to feed shrimps farmed in ponds in Thailand which are then exported to primarily urban global markets (Deutsch et al. 2007). Folke et al. (1997) estimated that people living in 744 large cities worldwide appropriate ~25 % of the globally available shelf, coastal, and upwelling areas for their seafood consumption. The connection of urban regions to globally dispersed areas of terrestrial production is illustrated by the global, spatial analysis of the link between plant production required for food, feed, fiber, and bioenergy supply and the location of the consumption of these products (Erb et al. 2009). It is not only land use related to the production but also implications of the water used to produce the food that is of concern. Globally, the volume of virtual water “embodied” in international food trade more than doubled in the period from 1986 to 2007 (Dalin et al. 2012).

**Fig. 1:**
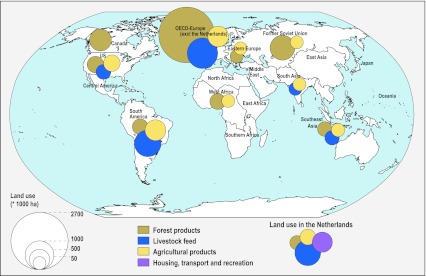
Land use for consumption in the Netherlands in 2000. Areas smaller than 50 000 ha are not shown (modified from Rood et al. 2004)

Studies of the urban metabolism of specific cities have documented the inflows, transformations, and outflows of resources and wastes (e.g., Warren-Rhodes and Koenig 2001; Kennedy et al. 2007). Ecological footprints of cities provide another approach. For example, an ecological footprint analysis of London indicated that around 80 % of food consumed in London is imported from other countries (Best Foot Forward Ltd. 2002 cited in Satterthwaite 2011). However, the geographic distribution of resource extraction and waste generation by individual cities is not yet available, although insights are provided by analyses of the global reach of resource use by highly urbanized countries such as The Netherlands and Japan. An analysis by Rood et al. (2004) documented the global distribution of land used by The Netherlands (Fig. 1). To supply the food and fiber needs of The Netherlands’ population, required an area four times larger than this small and highly urbanized country. This emphasizes the dependence on rural land and communities in other countries. The distal flows and connections between urban and non-urban regions are an important driver of land-use change (Seto et al. 2012). Some countries and corporations are now even attempting to assure their food and energy security via land lease arrangements in other countries (e.g., in Africa; Mbow 2010), which has impacts on land use as well as potentially negative and positive implications for local livelihoods.

As with many issues, land use does not stand alone but rather is interrelated with the use of other resources, including water and nitrogen. This is illustrated by the global analysis of the use of these resources in livestock production and trade (Galloway et al. 2007). For example, the consumption of meat (pork and chicken) in highly urbanized Japan is supported by the use in other countries (e.g., Brazil, USA, China) of over 2 million ha of land mainly for feed crop production, 3500 million m^3^ of water for irrigation and processing, etc., and 2.2 × 10^5^ metric tons of N fertilizer which contributes to aquatic eutrophication.

As the global urban population and its consumption increase, it is not only the sheer physical use of the planet’s resources, primarily from the hinterlands, that is of concern, but also the impacts on society and the environment. These impacts occur at many scales and the critical thresholds in many cases are crossed first at local and regional scales nearer the locus of resource use—with more immediate social and biogeophysical repercussions for regional food supply, water pollution as noted above, migration, social inequality, etc. For example, with increasing urbanization, emigration from rural areas to urban centers may not only erode rural communities but also continue to shift the focus of governments away from rural areas; this can lead to poor governance of the regions which are critical to the successful delivery of resource flows and ecosystem services to urban areas (Stafford Smith and Cribb 2009).

Given the complexity of systemic environmental and social issues now facing us, we should seek solutions that have positive, multiple synergetic effects and which, in combination, address the three dimensions of sustainability: social, economic, and environmental. Air pollution in many urban regions, including increasingly in Asia and Africa, poses major human and environmental health risks. At the same time a number of air pollutants also affect climate. To address the interrelated issues of climate and air pollution, Shindell et al. (2012) identified a suite of pollution-control measures. If these were to be implemented simultaneously with ambitious CO_2_ emission reductions, they suggest that global warming might be limited to <2 °C during the coming 60 years, with substantial direct co-benefits for human health and improved crop productivity.

Recent studies suggest that global food supply would need to roughly double by 2050 to meet the food and dietary changes of the primarily (~70 %) urban global population (Royal Society of London 2009; Godfray et al. 2010; UN 2011). Doubling global food supply without extensive additional environmental degradation to non-urban areas presents a major challenge (Foley et al. 2011; Tilman et al. 2011). Foley et al. (2011) suggested an approach to double food supply using a combination of measures to decrease the yield gap, decrease waste, and decrease meat consumption primarily in developed countries, while at the same time protecting key carbon sequestering ecosystems, biodiversity, and water quality. International co-operation in the form of technology transfer between rich and poor regions could be a key component of meeting food demands and at the same time reduce environmental degradation (Tilman et al. 2011). Technology transfer resulting in moderate intensification in croplands in under yielding nations could reduce, by 2050, land clearing by 80 %, land use-related GHG emissions by 1 Pg CO_2_-eq y^−1^, and N pollution of land and water.

In summary, the sustainability of a city can no longer be thought of in isolation from the combined resource use and impacts of cities globally. Urban areas are supported by human and natural resources often drawn from far distant regions. Multiple cities often draw on the same regions for their resource requirements. Therefore, interconnected solutions and new governance systems that take into account the planet’s limited resources are needed.

## Bringing Stewardship to Practice

Planetary stewardship must take into account the planet’s limited resources, interconnected issues, increasing urban population, and the reliance of urban areas on rural resources and their communities. Urban and rural are no longer useful boundaries to make with regard to planetary stewardship. It has become clear that urban activities drive much of the global changes we see, whether in energy use, resource depletion, land-use change, etc. Yet, we do not have adequate information on resource flows and their impacts or a conceptual framework for governance that takes into consideration the combined use of resources by cities and their interconnections with rural areas. At local scales efforts have been made to bridge the urban–rural divide and integrate social and ecological systems in regional urban planning (e.g., Alfsen et al. 2011). But how to address the planetary scale challenges?

**Fig. 2:**
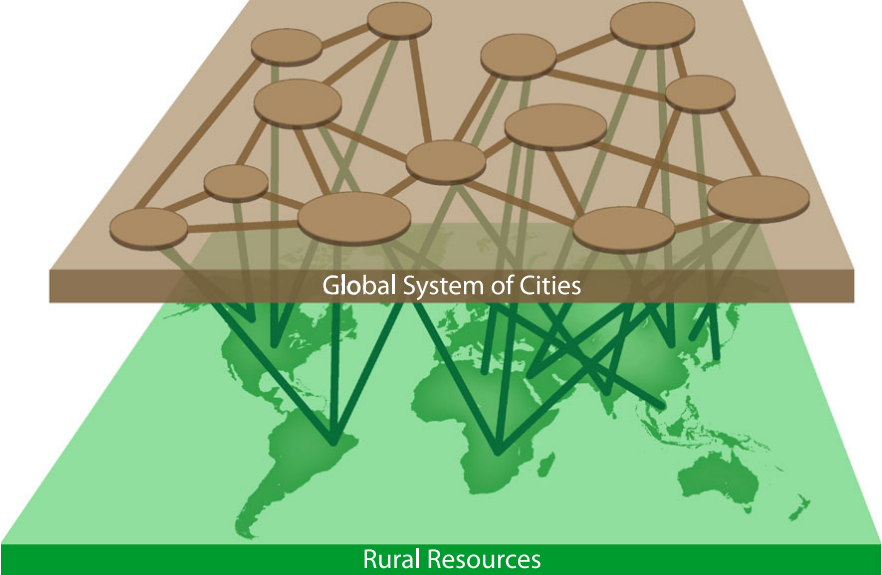
A global system of cities cooperating with rural regions for sustainable management of planetary resources

Many recent analyses have questioned the benefits of an exclusive reliance on a single global governance solution for tackling climate change and other environmental and socio-economic challenges (Ostrom et al. 1961; Biermann 2010; Ostrom 2010; Young 2011). The diverse and interconnected issues facing the planet warrant a cross-scalar, multi-agent approach to planetary stewardship. Because urban regions will likely remain key loci of intensive processing of global resources, they must take corresponding responsibility and that responsibility must connect to rural regions. In addition, the sustainability of an individual city must be seen within the context of the combined resource use by cities globally (Fig. 2).

Collaboration across a global system of cities could and should provide a new component of a framework to manage sustainable resource chains and their impacts (Fig. 2). The geographical and cultural diversity within a system of cities can provide powerful support for creative action (Ernstson et al. 2010; Olsson and Galaz 2012). However, sustainability practices and policies for a global system of cities must consider the urban teleconnections and therefore must be developed with a two-way dialog with distal rural areas. The potential for cities to cooperate as a system and with rural connectivity—as a positive component of the Anthropocene—could not only increase their capacity to effect change and foster stewardship at the planetary scale but also increase their resource security.

**Fig. 3:**
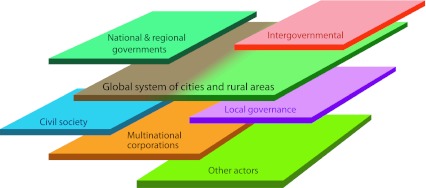
A collaboration across a global system of cities and rural areas must operate within a framework of actors at multiple scales

Cities are already engaging in cooperative partnerships and beginning to take an active role in the management of resources and impacts on the regional or even global scale. For example, complementary to national and international efforts to curb greenhouse gases, initiatives have emerged such as the C40 Cities Climate Leadership Group and the World Mayor’s Council on Climate Change. However, additional cooperative partnerships among urban and non-urban places are needed and these must extend to other global environmental issues, and address their interconnections and impacts on our planet. A global system of cities must also operate within a framework of other actors such as national, regional and local governments, multinational corporations, and civil society (Fig. 3). Each of these actors has important roles to play in managing planetary resources.

How to move forward given the magnitude and the complexity of the challenge, and insufficient knowledge, tools, and experience? Planetary stewardship of the sort proposed in this article is essentially untested. Experimental case studies that include cities across a range of geographic, development, and cultural settings are an essential first step. In addition, we suggest three priority areas of user-engaged research that are needed to bring planetary stewardship to practice. Co-design, co-production, and analysis of results by scholars, professionals, decision makers, and civil society should be a component in each of these.

1. Resources: Sustainable solutions require a deeper understanding of the geographic distribution of the planet’s resources, flows, interconnected uses, resultant wastes and stressors, and environmental and social impacts. The response of the social-ecological system to shocks (e.g., hurricanes, earthquakes, severe droughts) must be a component of such studies (Chapin et al. 2011). Studies should be developed within a fuller cost-accounting context considering the externalities of rural production and urban use. Building on existing and new knowledge a suite of user-friendly tools that allow analysis of future scenarios of resource use and impacts within a societal context should be developed.
2. Governance: We need empirical data on, for example, how the growing power and centrality of cities is appropriately connected to rural areas in terms of their empowerment and subsidiarity. This requires research on multi-dimensional networks that encompass different cities as well as the governance units along resource chains. Some specific questions to address include: what can facilitate better coordination between governance units at the same as well as different levels? How can polycentric governance increase resilience while at the same time minimizing the transaction and communication/coordination costs?
3. Information: Continuously updated information about coupled social-ecological systems is critical to achieve stewardship. Modern information technologies can support a system for monitoring and analysis of planetary conditions and support decision making at all levels. Putting this into practice will require sustainability services—an extension of the concept of the emerging climate services—to provide easy access to the data and analysis tools and a shared knowledge platform for communities of practice. At the same time, experimentation with novel models of governance will generate a pool of experience to draw on depending on the physical and socio-economic context.

Planetary stewardship that is mindful of society and the planet is the challenge of the Anthropocene. Effective stewardship must consider the multi-scale, interconnected resource chains, and their diverse actors. Urban regions must take an increased responsibility for motivating and implementing solutions that take into account their profound connections with and impacts on the rest of the planet.
